# Physical Education

**Published:** 1905-06-24

**Authors:** 


### Physical Education.

It is announced that the Lord Mayor will preside at a public meeting to be held at the Mansion House on Wednesday, the 28th instant, at 4.15 p.m., for the purpose of establishing a proposed National League for Physical Education and Improvement. The objects of the League will be (1) to stimulate public interest in the physical condition of the people throughout the kingdom; (2) to establish close association and centralisation of all societies and individuals trying to combat such influences as tend to produce national physical deterioration; (3) to aid existing organisations; and (4) to start organisations for physical health and well-being wherever none exists. It is further declared that the movement will be on strictly non-political and undenominational lines, and a suggested draft scheme of organisation on this basis, but subject to modification in various ways, has been put forward for consideration. As the purposes of the League are closely connected with medicine, the support of eminent physicians and surgeons in all three divisions of the United Kingdom has been enlisted.

As far as we can gather the intentions of the promoters from the sort of provisional prospectus which has been issued, it is intended to create an organisation extending to every part of the country, but developed, by means of local branches, in such a manner as best to supply the needs of each district in which such a branch is established. The efficacy of such a system would greatly depend upon the extent to which the local branches were rendered amenable to central control; without which they would be largely engaged in work of an experimental character, which might not have even the incidental utility of teaching others what to avoid. In such conditions, the voice of the faddist would speedily be heard to complain; and a diversity of administration, even greater than that of the local administration of the Poor Law, would be almost certain to arise. As shadowed forth in the provisional programme, the scheme casts a very wide net; and would include, for example, such different matters as the rearing of infants, the instruction of the rising generation in the first principles of cookery and of cleanliness, and the establishment of rifle ranges and of football clubs, as well as of facilities for the forms of athleticism best suited to the requirements of growing girls. To be effectual, any scheme for the rehabilitation of the working classes of this country must, we believe, be far more comprehensive than this. It must include all that is comprised in two almost forgotten words—in the first place thrift; in the second place the good old West country term "behaviour." Thanks to political and other causes, our labouring classes have gradually come into the possession of a larger amount of liberty and of independence than they have ever been taught to use wisely; and a perfectly relentless and merciless Nature is now enforcing penalties for the systematic violation of her laws. The late Mr. William Ellis preached in vain, for many years, on the text that the first duty of schools is to teach the principles of conduct; and the years during which that duty has been neglected are now bearing their bitter fruits in the consequences and conditions which we see around us. Mothers cannot suffer accumulations of filth within their dwellings, or stuff with rags every chink through which fresh air could obtain access to them, without laying up stores of provision for the sustenance of saprophytic bacilli. They cannot feed their children upon tinned meats and patent messes without reaping the harvest of rickets which naturally follows from such a seed-time. Working men cannot spend a large proportion of their wages in poisoning themselves with drugged beer and with tobacco, and at the same time provide proper sustenance for their families. School boards and County Councils cannot collect together great numbers of children, with a total neglect of proper sanitary supervision, without securing the occurrence and the extension of epidemics which, when they do not kill, leave behind the seeds of weakness and disease to undergo development in future generations. The causes of the evils which the new League proposes to combat must be looked for very deep down among the roots of many of our institutions. It is by dealing with the causes, and by this means only, that there can be any well-founded expectation of national physical amendment.

